# Hepatic angiosarcomatous transformation of a mediastinal germinal cell tumor: A care case report: Erratum

**DOI:** 10.1097/MD.0000000000015152

**Published:** 2019-03-15

In the article, “Hepatic angiosarcomatous transformation of a mediastinal germinal cell tumor: A care case report”,^[1]^ which appeared in Volume 96, Issue 51 of *Medicine*, the title appeared incorrectly and should be “Hepatic angiosarcomatous transformation of a mediastinal germinal cell tumor: A rare case report.”
